# Allergen Immunotherapy in Pediatric Asthma: A Pragmatic Point of View

**DOI:** 10.3390/children7060058

**Published:** 2020-06-08

**Authors:** Michele Miraglia Del Giudice, Amelia Licari, Ilaria Brambilla, Maria Angela Tosca, Giorgio Ciprandi

**Affiliations:** 1 Department of Woman, Child and of General and Specialized Surgery, University of Campania “Luigi Vanvitelli”, 80100 Naples, Italy; michele.miragliadelgiudice@unicampania.it; 2 Department of Pediatrics, Pediatrics Clinic, Ospedale San Matteo, University of Pavia, 27100 Pavia, Italy; a.licari@smatteo.pv.it (A.L.); i.brambilla@smatteo.pv.it (I.B.); 3 Department of Pediatrics, Allergy Center, Istituto G. Gaslini, 16100 Genoa, Italy; mariangelatosca@gaslini.org; 4 Allergy Clinic, Casa di Cura Villa Montallegro, 16100 Genoa, Italy

**Keywords:** allergen-specific immunotherapy, subcutaneous immunotherapy, sublingual immunotherapy, allergic asthma, children

## Abstract

To date, the only disease-modifying treatment strategy for allergic rhinitis and asthma is allergen immunotherapy (AIT). There is evidence that AIT improves allergic rhinitis and asthma, such as reducing symptom severity and medication use and improving of quality of life, with a long-lasting effect after the end of the course. The recent clinical trials evidenced AIT effectiveness and safety in allergic asthma. Consequently, the current version of the GINA (Global Initiative for Asthma) guidelines recommend AIT as an add-on therapy for asthma. There is also evidence that AIT may exert preventive activity on the possible progression from allergic rhinitis to asthma in children and the onset of new sensitizations. The present review provides a pragmatic summary of the clinical indications of AIT in pediatric asthma, including the immunological mechanisms, the predictive biomarkers, and the safety issues in clinical practice.

## 1. Introduction

At present, allergen-specific immunotherapy (AIT) remains the only curative treatment of allergic disorders. This therapeutic approach uses administration of the causal allergen to improve specific IgE-mediated response, thereby controlling symptoms. AIT has been used for over a century. AIT is still the only disease-modifying treatment strategy for allergic diseases, as it induces a long-lasting immunological and clinical tolerance toward the causal allergen [1]. For this reason, the most important worldwide regulatory authorities, such as the Food and Drug Administration (FDA) and European Medicines Agency (EMA), have approved AIT.

Subcutaneous immunotherapy (SCIT) and sublingual immunotherapy (SLIT) are the most used and accepted routes of administration and are effective for both adults and children with respiratory allergies such as allergic rhinitis (AR) and asthma [2,3]. SCIT was initially employed with the first description by Leonard Noon [4]. However, SCIT is still limited by the need for frequent injections over a minimum of 3 years, periodic visits to the doctor′s office, and, overall, the potential occurrence of severe systemic reactions. Consequently, there is agreement about the practice that SCIT should only be administered in an adequate medical setting, and clinicians should be trained to manage anaphylactic side effects [1]. The risk of SCIT systemic reaction is higher in uncontrolled asthma and with rush schedules [5]. Therefore, SLIT could be a reliable alternative to SCIT, mainly in children, as, at home, self-administration is possible, and severe systemic reactions are reduced [6,7,8]. In clinical practice, the route choice depends on several factors, including product availability or approval, geographic location, cost, patient′s characteristics, physician attitude, and patient preference [9].

The current products for SCIT or SLIT cannot be compared because of their heterogeneous composition and allergen concentration given by different manufacturers [10].

There is evidence that both SCIT and SLIT induce superimposable immunologic effects [11]. Rough allergen extracts are used for SCIT, and there are also chemically modified allergens (allergoids). SLIT is available as an aqueous solutions or tablets. Pending harmonized and international rules to regulate AIT products, there are two situations: distribution as “named patient products” (NPP), which only require to be prepared in compliance with Good Manufacturing Practice, or formal marketing authorization [12]. According to EMA directives, SLIT tablets for grass pollen and house dust mites (HDM) have been approved [13,14].

Thanks to mechanisms providing allergen tolerance, effective AIT may affect the natural history of allergy, preventing new sensitizations and clinical worsening, such as a progression from allergic rhinitis to asthma. Moreover, AIT controls allergic symptoms when not responsive to avoidance or pharmacotherapy, reduces medication use, improves the quality of life, and has long-lasting effects after the end of treatment [15]. The most recent clinical trials strengthened the evidence concerning the AIT effectiveness and safety to treat allergic asthma with SLIT as an add-on therapy [3]. Furthermore, AIT has effects preventing asthma in AR subjects, mainly if started early in childhood [16].

## 2. Overview of the Mechanisms of Allergen Immunotherapy

The targets of AIT, including both SCIT and SLIT, are the immune response consequent to the causal allergen exposure. Modulating immune response, AIT regulates T- and B-cell changes, immunoglobulin class decreases mediator release, and migration of eosinophils, basophils, and mast cells to inflamed tissues [17,18,19].

AIT-induced immunologic tolerance is based on the upregulation of allergen-specific T-regulatory (Treg) cells and B-regulatory (Breg) cells, and the consequent down-regulation of the T helper 2 (Th_2_) response [19] (Table 1).

Treg and Breg cells produce interleukin (IL)-10 and transforming growth factor-β (TGF-β), regulatory cytokines inhibiting the activation of allergen-specific Th_2_ lymphocytes, suppressing type 2 inflammation, and ultimately shifting toward a physiological Type 1-mediated immunity [20].

High-dose AIT induces many immunological modifications: dendritic cells (DCs) produce IL-12, IL-27, and IL-10, which generate and activate distinct phenotypes of Treg cells, in particular, forkhead box P3 (Foxp3)^+^ Treg and inducible Treg (iTreg) cells [17,19]. Both Foxp3^+^ Treg and iTreg cells suppress allergic reaction, releasing regulatory cytokines (IL-10, TGF-β, and IL-35), inducing tolerogenic DCs subsets, suppressing allergen-specific Th_2_ lymphocytes, downregulating the expression of FCεRI receptors on mast cells, decreasing allergen-specific IgE, and promoting IgG_4_ synthesis [17,19,20,21,22]. IL-10 exerts inhibition of IL-4 and IL-5, allergen-specific IgE, but increases IgA and IgG_4_ [19]. The competitive effect of IgG_4_ toward IgE antibodies is an “immunologic blockade” that inhibits mast cell and basophil degranulation. TGF-β suppresses Th_2_ and innate lymphoid cells type 2 (ILC2), thus reducing type 2 inflammation [17,19].

Breg cells promote allergen immune tolerance, by producing IL-10 and TGF-β, blocking IgG_4_, inhibiting effector T cells, suppressing type 2 inflammation, and restoring allergen-specific Treg cells [21].

## 3. Clinical Indications of Allergen Immunotherapy in Children with Respiratory Allergy

AIT is indicated in patients suffering from AR with or without conjunctivitis, and/or asthma, after documenting a true allergy to the causal allergen [22]. Candidates for AIT are patients whose symptoms are not controlled adequately by medications and environmental measures or those experiencing unacceptable adverse effects of medications or who wish to reduce the long-term use of medications [23,24,25].

There is evidence that AIT is effective, safe, and preventive in AR and/or asthma in adults and children, even though there is high heterogeneity among the study populations enrolled, the different schedules, the different AIT products, and the outcomes [6,23,24,25,26,27]. As SLIT is more manageable than SCIT, it has to be preferred in children.

## 4. Children with Asthma

The prevalence of asthma is about 5–10% in childhood and adolescence [28]. The allergic asthma phenotype is characterized by type 2 inflammation, airway hyperresponsiveness (AHR), and reversible airway obstruction [29]. Asthma control is currently considered the aim of asthma therapy. Asthma management is control-based, such as on a stepwise approach, and gradually titrated [30]. Asthma control is defined as the level to which asthma symptoms are changed by therapy [31,32,33]. However, it has to be underlined that standard pharmacotherapy is merely symptomatic because it does not affect the underlying pathogenetic mechanisms, as, after its cessation, symptoms and inflammation occur again.

SLIT is recommended as an add-on treatment option in adult asthmatics sensitized to HDM, with comorbid AR, and having exacerbations despite inhaled corticosteroid (ICS) treatment, with forced expiratory volume in 1 s (FEV_1_) more than 70% predicted, as stated in the latest Global Initiative for Asthma Report (GINA) update [3]. The inclusion of SLIT in HDM was based on a meta-analysis that documented SLIT efficacy in patients with asthma [34]. Moreover, asthma has to be carefully managed throughout the AIT course to prevent uncontrolled asthma [35]. On the other hand, AIT is contraindicated in uncontrolled asthma, as patients with uncontrolled asthma may experience severe adverse reactions [5]. In this context, adding anti-IgE biological therapy (omalizumab) could be a suitable option for increasing the effectiveness and the safety of AIT, particularly in SCIT [35,36,37,38].

This significant change in the GINA asthma management strategy draws upon recently published results from a Phase III clinical trial. It evaluated the treatment of HDM allergic asthma with the standardized quality (SQ) HDM SLIT tablet in adults: the addition of HDM SLIT to maintenance therapies improved the requirement for ICS or the time to first exacerbation upon ICS reduction, suggesting that SQ HDM SLIT-tablet treatment may contribute to improving overall asthma control [39,40]. While these data require further studies to assess the long-term efficacy and safety of SQ HDM SLIT in adults, less information is available for adolescents [41,42], and studies are still in progress in children less than 12 years of age [43]. HDM sensitization in early childhood has been identified as an important risk factor for the development of asthma later in childhood and is associated with impaired lung function in school-age children [44] and persistence of asthma into adulthood [45]. Moreover, sublingual treatment, using 300 index of reactivity (IR)-standardized HDM extract, resulted in improved rhinitis and/or asthma symptoms scores in children aged 6–18 years with AR with or without allergic asthma [46].

There is convincing evidence about the efficacy of AIT in adults and children with allergic asthma, as confirmed by meta-analyses, mainly concerning the impact of SLIT in asthmatic children, even though there were variable results due to the heterogeneity of tested products and clinical outcomes [22,27,47,48,49,50,51,52,53,54]. Combined AR and asthma symptom and medication scores significantly diminished in asthmatic children with comorbid AR after SLIT treatment [48,53,55]. Symptom improvement, reduction in medication use, hospitalizations, emergency department visits, and school absences also persisted after AIT discontinuation. There is evidence that a prospective study showed fewer asthma episodes, the use of relievers, and improved lung function in asthmatic children after 5 years of AIT discontinuation [56]. SCIT was able to control asthma and reduce medication use, including relievers and controllers [49,50,57]. Significant reduction in asthma symptoms, asthma medication use, and airway hyperreactivity in children and adults was reported by meta-analysis [49,50]. These outcomes were confirmed by more pediatric studies [58,59,60,61]. SCIT exerts a long-term impact on childhood asthma, as, after a 3-year SCIT, a global remission of asthma was obtained [62]. Moreover, children re-evaluated 9 years after SCIT discontinuation, had a three times lower risk of frequent asthma symptoms than controls [63].

SCIT and SLIT are, therefore, effective in treating AR and asthma in children [64,65]. Notably, AIT could reduce ICS doses, guaranteeing asthma control. AIT added to ICS maintenance therapy reduced ICS doses while still maintaining asthma control in up to 42% of patients [41,61].

## 5. Patient Selection and Biomarkers of Response

It has to be underlined that AIT is allergen-specific; therefore, a careful workup is mandatory [2]. Major allergens should be identified by molecular diagnostics [66,67,68]. Benefits and risks, and the ability to comply/cooperate with AIT, should always be assessed [26]. AIT was effective and safe in preschool children [69,70], but there is no agreement about a specific lower age limit [8].

On the other hand, it is a collective experience that some patients are non-responders to AIT. Therefore, the identification and validation of potentially predictive biomarkers of response to AIT is an active field of research. Biomarkers are quantitative measurements that predict clinical and immunological effects of AIT [18,71] The list includes cellular (Tregs), humoral (allergen-specific IgG_4_ (sIgG_4_), IgE/IgG_4_), molecular (interleukins), and functional (IgE FAB and blocking factor) biomarkers. However, in clinical practice, very few biomarkers are reliable (Table 2). There is evidence that higher levels of sIgE could help predict response to AIT efficacy [72,73,74].

Regarding the monitoring of AIT efficacy, it has to be considered that the combined assessment of symptom severity and medication use is the most reliable tool [75]. In particular, the visual analog scale could be an easy way to measure the patient′s perception of AIT improvement [76].

As SLIT may be self-administered and is well tolerated, it is more indicated in children.

## 6. Safety Issue

The safety of AIT has always been a crucial issue in all clinical trials. Many aspects should be considered in clinical practice—an EAACI position paper listed the contraindications to AIT [5]. Moreover, a series of recommendations should carefully comply in clinical practice, as reported in many official documents [77,78,79]. If AIT is prescribed and administered correctly and prudently, AIT, mainly SLIT, is safe and well-tolerated also in children with allergic asthma. In this regard, SLIT may induce local side effects, including oral itching and swelling, usually self-resolving, in about 30–40% of subjects [77]. Systemic adverse reactions are infrequent in patients treated with SLIT. Therefore, SLIT is preferable in children.

## 7. New Perspectives

Although AIT is a consolidated therapeutic option, there is room for further insight and investigation in the future. First, as the immune system is plastic in children, early AIT could prevent natural allergy history progression. In this regard, the GAP (Grazax asthma prevention) study provided evidence that five-year SLIT prevented asthma onset in children with allergic conjunctivitis, and, overall, AIT was more effective in younger children [80]. This outcome underlined the concept that AIT should be considered a disease modifier and should be prescribed as soon as possible. The real prevention should be timely. A second issue is a possible synergy with biologics, namely omalizumab. Even though biologics are still indicated in severe asthma, it is conceivable that a high dose of the causal allergen could increase the production of IgE and so raise the risk of an adverse event.

As a consequence, biologics could improve the safety profile of AIT [81,82]. Another aspect is the possible use of IgG_4_ as a biomarker for assessing the response to AIT. IgG_4_ is a useful marker of B_reg_ activity and is also commercially valuable. Therefore, serum IgG_4_ assay could support the clinical evaluation of the AIT response. Last but not least, adherence to AIT is a critical issue, as many patients discontinue this therapy. Different factors can influence this issue, including high cost, duration, frequent medical visits, and poor perception of quick symptom relievers. Patient engagement could be an attractive way to improve AIT adherence [83].

## 8. Conclusions

AIT is a valuable therapeutic option, especially in childhood, to modify the progression of respiratory allergic disease [84,85]. Both SCIT and SLIT are useful in pediatric allergic asthma, having an intriguing steroid-sparing effect. However, uncontrolled asthma must be a strong contraindication for AIT treatment. However, coupling novel biological therapies with AIT could represent a promising approach to potentially avoiding adverse reactions. At present, AIT should be considered the best expression of personalized medicine in clinical allergy.

## Figures and Tables

**Table 1:** Mechanisms of immunologic tolerance mediated by T and B regulatory cells during allergen immunotherapy.

**Treg-Mediated Mechanisms**
release regulatory cytokines (IL-10, TGF-β, and IL-35)
induce tolerogenic DCs subsets
reduce number of ILC2
suppress activation of allergen-specific Th_2_ lymphocytes
downregulate the expression of FCεRI receptors on mast cells,
decrease allergen-specific IgE synthesis
promote B-cell production of IgG_4_ antibody
**Breg-Mediated Mechanisms**
release regulatory cytokines (IL-10, TGF-)
induce the synthesis of IgG4 blocking antibodies
inhibit activation and proliferation of effector T lymphocytes
suppress Th_2_-dependent inflammation
promote T-cell expression of Foxp3 and generation of functional Treg cells

Breg, B regulatory; DCs, dendritic cells; FCεRI, high-affinity receptor for the Fc region of IgE; Foxp3, forkhead box P3; IgE, immunoglobulin E; IgG_4_, immunoglobulin G subtype 4; IL, interleukin; ILC2, innate lymphoid cells type 2; TGF, transforming growth factor; Th_2_, T helper type 2; Treg, T regulatory.

**Table 2:** Pragmatical biomarkers for allergen immunotherapy (AIT).

Categories	Candidate Biomarkers	Possible Applications
Biomarkers for diagnosis	Allergen-specific IgE	Prediction of disease severity and/or progression
Biomarkers for response prediction	Allergen-specific IgE (high levels)	Prediction of AIT response
Biomarkers of AIT efficacy	Symptom score Medication use	Prediction of patients’ response
